# Evaluation of Physicochemical Properties of Polymeric Systems for Potential Applications in Cartilage Tissue Engineering

**DOI:** 10.3390/ijms26052057

**Published:** 2025-02-26

**Authors:** Dominika Wanat, Claudia Garbowska, Wiktoria Wrzesińska, Oliwia Grzywacz, Katarzyna Sala, Kacper Zapotoczny, Magdalena Bańkosz, Josef Jampilek, Janusz Walter, Bożena Tyliszczak

**Affiliations:** 1 Department of Materials Engineering, Faculty of Materials Engineering and Physics, CUT Doctoral School, Cracow University of Technology, 37 Jana Pawla II Av., 31-864 Krakow, Poland; dominika.wanat@doktorant.pk.edu.pl (D.W.); magdalena.bankosz@pk.edu.pl (M.B.); 2 Department of Materials Engineering, Faculty of Materials Engineering and Physics, Cracow University of Technology, 37 Jana Pawla II Av., 31-864 Krakow, Poland; claudia.mordaka@student.pk.edu.pl (C.G.); wiktoria.wrzesinska@student.pk.edu.pl (W.W.); oliwia.grzywacz@student.pk.edu.pl (O.G.); katarzyna.sala@student.pk.edu.pl (K.S.); kacper.zapotoczny@student.pk.edu.pl (K.Z.); janusz.walter@pk.edu.pl (J.W.); 3 Department of Analytical Chemistry, Faculty of Natural Sciences, Comenius University, Ilkovicova 6, 842 15 Bratislava, Slovakia; 4 Department of Chemical Biology, Faculty of Science, Palacky University Olomouc, Slechtitelu 27, 779 00 Olomouc, Czech Republic

**Keywords:** hydrogels, cartilage tissue, photopolymerization, regeneration systems

## Abstract

This study investigates the physicochemical properties of hydrogels based on PVA and PVP crosslinked with PEGDA, focusing on their swelling capacity, surface roughness, incubation behavior, and structural modifications upon bioactive component incorporation. Swelling analysis demonstrated that the amount and molecular weight of PEGDA significantly influences the hydrogels’ sorption properties, with the highest swelling coefficient observed for samples with 2 mL PEGDA (575 g/mol) due to a looser network structure, while the lowest was recorded for 2.5 mL PEGDA (700 g/mol), indicating a denser network. Surface roughness analysis revealed that increasing the crosslinker amount led to higher roughness both before and after incubation, with samples containing 575 g/mol PEGDA being more susceptible to structural changes in an incubation environment. FT-IR spectroscopy confirmed the presence of characteristic functional groups, providing insight into the chemical stability and hydration properties of the hydrogels. Modification with a bioactive mixture (glucosamine, chondroitin, and MSM) was confirmed by spectral analysis, indicating successful integration without compromising the hydrogel matrix. The modified hydrogels demonstrated potential applications in regenerative medicine, particularly for joint disease treatment and cartilage tissue repair.

## 1. Introduction

Biomaterials engineering is of significant interest to scientists and researchers. The field continues to grow and is widely used in the development of both new medical devices and new biomaterials with more efficient properties [1]. A hydrogel is characterized by a porous three-dimensional (3D) structure composed of hydrophilic polymers that can swell, effectively accommodating high water content. Swollen hydrogels are supported by chemical or physical cross-linking of the polymer chains, providing structural integrity [2,3]. Synthetic polymers, unlike natural ones, have higher mechanical strength and the ability to precisely control the rate of biodegradation. However, the process of synthesizing synthetic polymers can lead to hydrogel toxicity, resulting in the induction of an immune response and rejection after application to wounds. To alleviate these concerns, researchers are investigating mimicking the natural structure of the polymer and enhancing its functionality while developing new hydrogels based on synthetic polymers [4]. PVA/PVP hydrogels are composed of two synthetic, non-ionic, homopolymer chains that undergo physical or chemical cross-linking [5]. The structural properties and wetting ability of hydrogels can affect healing, leading to faster tissue regeneration. Therefore, in recent decades, research has focused on synthesizing nontoxic hydrogels and testing their degree of wetting, hydration, and swelling, as well as their stability under different temperature and humidity conditions [6]. In order to reduce the biological inertness of PVA/PVP hydrogels and increase cell adhesion, Jalageri et al. mixed PVA and PVP with hydroxyapatite nanorod (HNr), and the hydrogel synthesis was carried out by a freeze–thaw process. This significantly increased the elastic modulus, which was close to that of cartilage, indicating promising potential for use as a next-generation cartilage substitute [7]. Himawan et al. synthesized a PVA/PVP hydrogel with citric acid, crosslinking the composite at 80 °C. The resulting hydrogel has acceptable mechanical strength, and the hydrogel patch can act as an absorbent for the hydrophilic drug contained in the skin but can also provide it [8]. Poly(vinyl alcohol) (PVA) is a synthetic polymer that has a hydroxyl group in its structure. It is in high demand due to its diverse properties and wide applications in various fields. It has been one of the most important synthetic polymers produced globally for more than a century. Its biocompatibility and ability to release active substances in a controlled manner make it an attractive material for biomedical applications. However, studies have shown that PVA-based biomaterials have certain properties that may limit their application or performance. To overcome these, various methods have been reported, among which blending with poly(vinylpyrrolidone) (PVP) has shown promising results [9]. One of the key features of PVP is its ability to form complexes with other substances, making it an excellent carrier for various active substances, such as drugs and dyes. PVP is a water-soluble polymer that exhibits no toxic effects, used as an excipient in pharmaceuticals, as a blood plasma substitute, and as a dressing in skin regeneration. Hydrogels based on the synergy of poly(vinylpyrrolidone) (PVP) and poly(vinyl alcohol) (PVA) have a high water absorption capacity, making them ideal for applications in hydration and maintaining the right environment in areas such as skincare and burn treatment. Hydrogels made from a mixture of PVA and PVP have been shown to exhibit better stability and less degradation due to the inter-chain hydrogen bonding between hydroxyl groups on PVA chains and carbonyl groups on PVP chains. In addition, their flexibility and biocompatibility make them potentially useful as drug carriers, including for the controlled release of active ingredients. An attempt to overcome the limitations of PVA was also made by Sun et al., who used PVA to obtain multifunctional sensors. By adding ethylene glycol (EG), sodium alginate (SA), and ZnSO_4_ to PVA, they increased the stability of the hydrogels and their accuracy for use as dual-network sensors. The resulting ability to detect deformation and pressure enables the sensors to be used in both underwater and airborne environments [10]. The polymers used to obtain hydrogels find various types of applications. Buratti et al. used the PEGDA polymer with the addition of silver nanoparticles to print three-dimensional hydrogels that act as a water filter to remove mercury ions [11]. In turn, Wojcik-Pastuszka et al. used sodium hyaluronate (HA) to obtain hydrogels released from certain drugs selected by the authors. They proved that the concentration of the biopolymer affects the efficiency of the drug release mechanism [12]. Chen et al. focused on the potential applications of hydrogel dressings for diabetic wound healing from the perspective of clinical applications. They discussed the environment of this type of wound, as well as the advantages and prospects for the development of hydrogel dressings [13]. For this, Cao et al. in their article presented a systematic review of the correlations between the physicochemical properties, composition, and structural features of hydrogels and cell biology and related signaling cascades, as well as the application of current advances in hydrogels for biomedical applications [14]. Volpi et al. presented tissue engineering of skeletal muscle-based scaffolds and hydrogel-based microfibers that provide a platform to support skeletal muscle tissue development [15].

This study investigates the physicochemical properties of hydrogel materials based on polyvinyl alcohol (PVA) and polyvinylpyrrolidone (PVP). The evaluation encompassed the analysis of swelling capacity, surface roughness profile, chemical composition determined through FT-IR spectroscopy, and stability during incubation in simulated body fluids. The hydrogels were prepared using crosslinking agents with varying molecular weights and concentrations, enabling an assessment of how these parameters influence the materials’ properties. The selection of PEGDA as the crosslinking agent, with molecular weights of 700 g/mol and 575 g/mol, was based on the assumption that varying the molecular weight could influence the network structure of the hydrogels, thereby affecting properties such as swelling capacity and stability in physiological conditions. These properties are considered important for potential applications in cartilage tissue regeneration. By comparing hydrogels prepared with different molecular weights of PEGDA, the study aimed to evaluate how the crosslinking density might impact the physicochemical characteristics of the resulting materials.

The results demonstrated that the obtained hydrogels exhibited high chemical and structural stability under simulated biological conditions. Subsequent modification of the hydrogels involved incorporating a mixture of bioactive compounds, including chondroitin, glycosaminoglycans, and methylsulfonylmethane (MSM), which endowed the materials with additional biological properties, potentially enhancing tissue regeneration processes. The innovation of this work lies in its comprehensive approach to designing hydrogels with tunable structural and biological characteristics tailored for specific biomedical applications. The use of different crosslinking agents and the incorporation of bioactive components enabled the creation of functional materials with potential applications in tissue engineering and other areas of regenerative medicine.

## 2. Results

### 2.1. Analysis of Sorption Capacity

**Figure 1:**
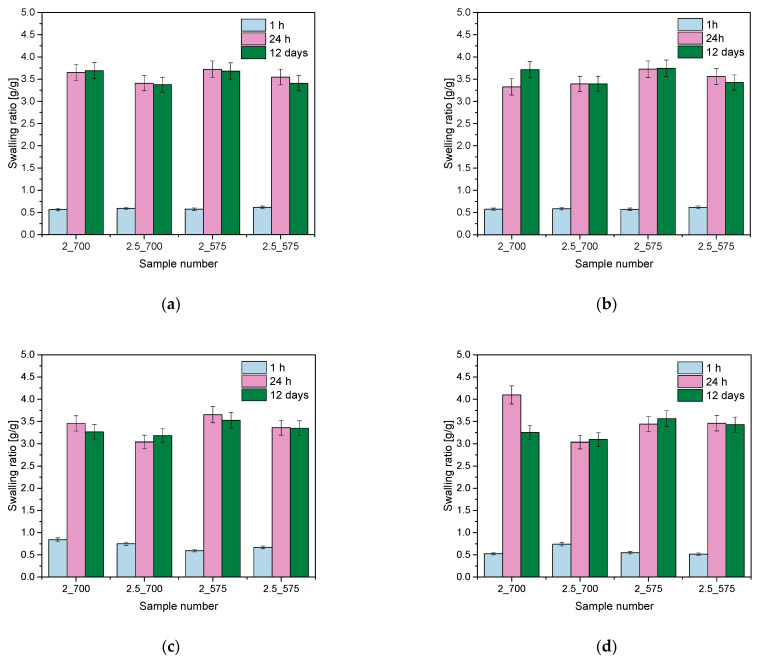
Results of sorption capacity analysis in distilled water (**a**), citric acid (**b**), Ringer’s solution (**c**), and saliva (**d**).

To investigate and compare the sorption capacity of the hydrogels, a swelling analysis was conducted. The study considered the effect of the amount of PEGDA crosslinking agent added and the addition of PVP and PVA. The results of the analysis of citric acid, saliva, Ringer’s solution, and distilled water are shown in the graphs below (Figure 1).

In order to investigate and compare the sorption capacity of the hydrogels, a swelling analysis was performed. The study considered the effect of the amount of the PEGDA cross-linker added. The analysis results for citric acid, saliva, Ringer’s solution, and distilled water are presented in the graphs above (Figure 1). The tested hydrogel materials showed the ability to bind liquids, with the fastest increase in the swelling coefficient observed in the first 24 h, after which the values stabilized at similar levels. The swelling coefficients ranged from 0.5 g/g to over 4 g/g, with the lowest value obtained for samples containing 2.5 mL of PEGDA cross-linker with a molecular weight of 700, which may suggest that a higher concentration of this agent limits the ability of the hydrogels to absorb water. The sample containing 2 mL of PEGDA with a molecular weight of 575 g/mol had the highest swelling ratio in all the fluids analyzed, which can be explained by the lower amount of crosslinking agent and lower molecular weight, leading to a looser hydrogel structure that promotes liquid absorption. An interesting finding was the observed sudden jump in the sorption properties of the sample with 2 mL of 700 g/mol PEGDA in the artificial saliva, which deviated from the general trend. This may be due to the interaction of specific components of artificial saliva with the hydrogel network, such as ions or other components that may affect the network structure or its ability to bind water. In other liquids, such as Ringer’s fluid, citric acid, and distilled water, this effect was not observed, confirming that the sorption properties of the hydrogel may be strongly dependent on the chemical composition of the medium. Similar results were obtained by researchers in experiments on PEGDA hydrogels, where biological environments such as phosphate buffer (PBS) contributed to higher swelling rates compared to distilled water, indicating the importance of the complex chemical composition of the solution in stimulating water absorption [16]. Other studies have demonstrated the key effect of the hydrogel manufacturing method on its swelling ability. It was found that the porous structure of hydrogels, obtained using 3D printing techniques, promotes higher swelling rates, even in environments with complex chemical compositions, such as physiological solutions. These results indicate that the hydrogel parameters can be further tuned depending on the requirements of the application environment [17].

### 2.2. Microscopic Observations with Determination of Roughness Profile

**Figure 2:**
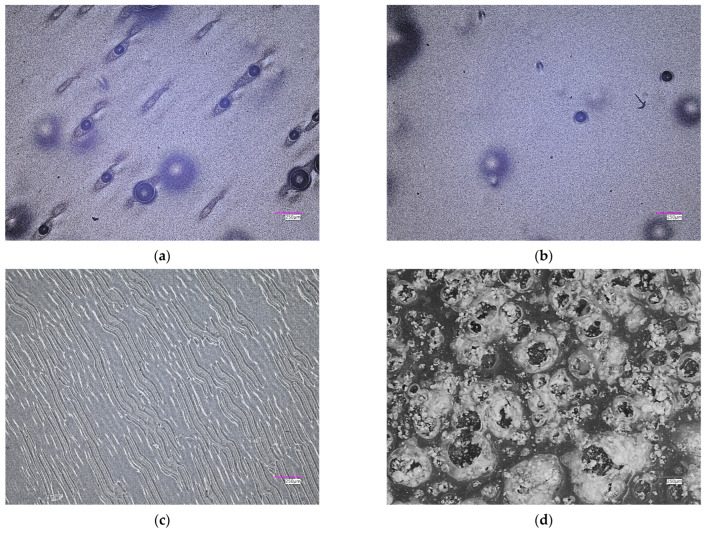
Observations with a digital microscope, magnification 100×. Sample name 2_575 (**a**); 2.5_575 (**b**); 2_700 (**c**); 2.5_700 (**d**) (photos of dry samples not incubated).

**Figure 3:**
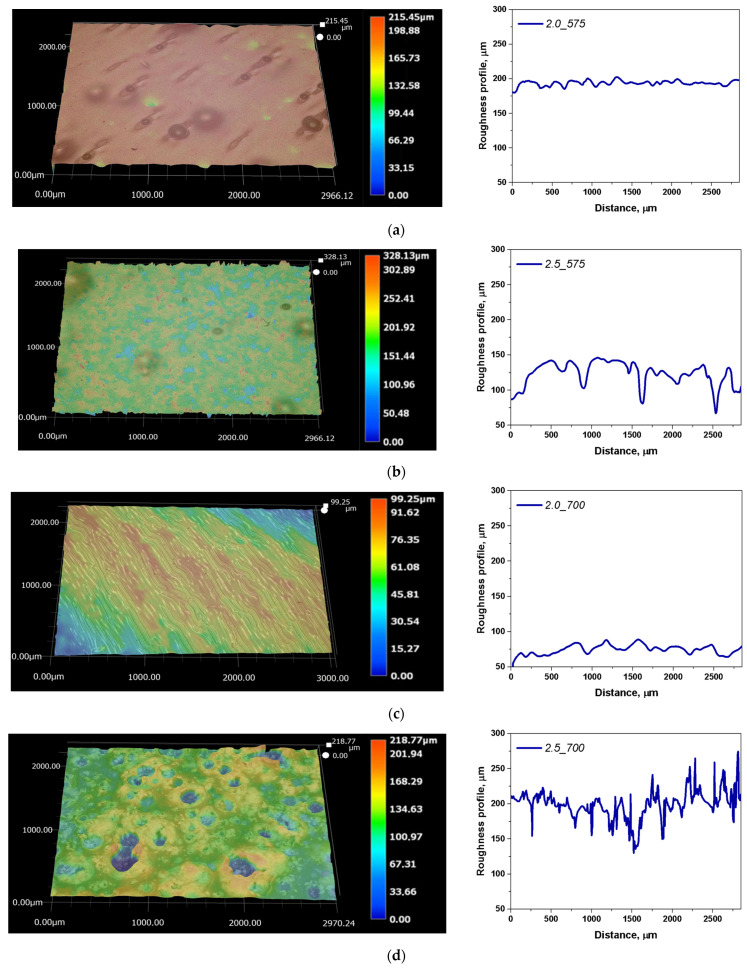
View of surface and roughness profile: 2_575 (**a**); 2.5_575 (**b**); 2_700 (**c**); 2.5_700 (**d**) (on the left, a 3D view of the hydrogel material, and on the right, the corresponding roughness profile).

Microscopic observations in the report with the determination of the surface roughness profile were taken into account concerning the structures of hydrogel matrices. The results of the microscale surface morphology and texture analysis are available in Figure 2 and Figure 3. A summary of the key parameters for the performed analyses is summarized in Table 1.

As part of the study, parameters such as Ra (the arithmetic mean deviation of the profile height from the mean line) and Rz (the maximum profile height) were determined. The surface roughness analysis results for the hydrogels showed that the amount of crosslinking agent (PEGDA) significantly influences the surface properties of the materials. The presented findings indicate considerable variability in both parameters across all tested samples. A comparison of samples containing 2 mL and 2.5 mL of PEGDA revealed a clear relationship between the increased amount of the crosslinking agent and greater surface roughness. The Ra values ranged from 2.43 µm to 14.32 µm, indicating differences in surface smoothness, while the Rz values ranged from 17.42 µm to 153.47 µm, reflecting significant differences in surface topography. The sample containing 2 mL of PEGDA with a molecular weight of 575 g/mol exhibited the lowest Ra and Rz values, suggesting a homogeneous surface. The roughest surface was observed for the sample containing 2.5 mL of PEGDA with a molecular weight of 575 g/mol, showing the highest Rz value (153.47 µm) and significantly elevated Ra (14.32 µm) compared to other samples. These parameters suggest an uneven surface with pronounced peaks and valleys on the profile graph. Samples with a higher degree of crosslinking, i.e., with a higher PEGDA content (2.5_575; 2.5_700), showed significantly higher Ra values, resulting in a more irregular surface. The maximum differences in Rz values were also observed in polymer matrices with higher crosslinking degrees. This relationship was most evident for the sample containing 2.5 mL of PEGDA with a molecular weight of 575 g/mol, which exhibited the roughest surface. In general, increasing the amount of crosslinking agents led to an increase in the surface roughness of the tested polymer matrices. Based on the results obtained and detailed analysis, no significant changes in Ra and Rz parameters were observed concerning the molecular weight of the crosslinking agent, but rather concerning the amount of PEGDA used. The quantity of the crosslinking agent influences the formation of the hydrogel structure. The use of a higher amount of PEGDA results in a more complex and densely crosslinked structure, affecting the physicochemical properties and surface topography of the hydrogels.

### 2.3. Results of the Incubation Study

**Figure 4:**
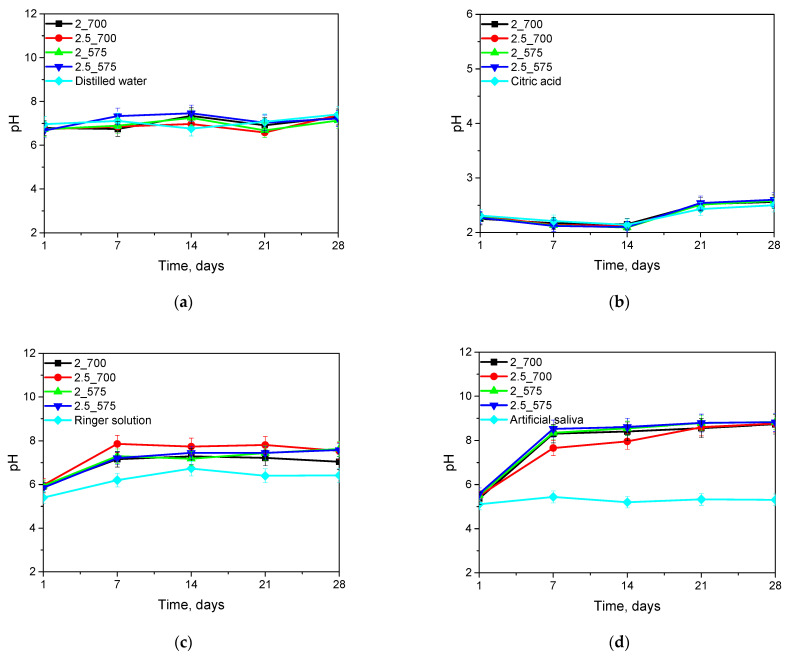
Results of incubation analysis in distilled water (**a**), citric acid (**b**), Ringer’s solution (**c**), and saliva (**d**).

Incubation tests were performed to determine the hydrogen ion activity (pH) in the liquid in which the hydrogel matrices were immersed for 28 days. The results of these tests are shown in the graphs below (Figure 4).

The incubation study is important because we know how the hydrogel material interacts with the environment. Changes in pH value may indicate the interaction of the hydrogel with incubation fluids. The release of substances or chemical reactions is important in the context of the use of matrices for the biomedical industry. The conducted studies show that significant pH jumps do not occur for most incubation fluids, except artificial saliva. The incubation graph of the tested sample in artificial saliva is shown in Figure 4c. A pH jump to the alkaline side is visible. Artificial saliva has a pH of 5, while after incubation, the level of the tested fluid changes to a value between 8.5 and 9 pH. The smallest changes in pH value are observed for incubation in distilled water and citric acid. These two incubation fluids did not affect the release of ions and the degradation of the incubated materials. An interesting result is also obtained for the sample 2.5(700) incubated in Ringer’s solution, which had the largest amount of cross-linking agent with a higher molecular weight. It is clearly visible that the release of ions occurred the fastest and the pH result in this tested sample reached the highest result, namely pH at the level of 7.5–8. An important aspect of the analysis was the temperature at which our samples were stored over time. The samples were incubated at 37 °C. We did not observe drastic changes in pH during the incubation of the samples in distilled water, citric acid, or Ringer’s solution, which indicates the lack of degradation of the hydrogel matrices, but only the release of active substances, whereas a drastic jump in pH was observed when testing the sample incubated in artificial saliva, which also did not affect the degradation of the tested sample. Similar results were obtained by Schüler et al. They studied the effect of various buffers, including saliva, on the degradation of hydrogel composites. They proved that there was no degradation of the material despite a large pH jump [18]. Fan et al. also studied the effect of incubation on sample degradation in artificial saliva, but their results jump to the effect of incubation on the degradation of the tested matrices [19].

### 2.4. Results of Infrared Spectroscopy Analysis

**Figure 5:**
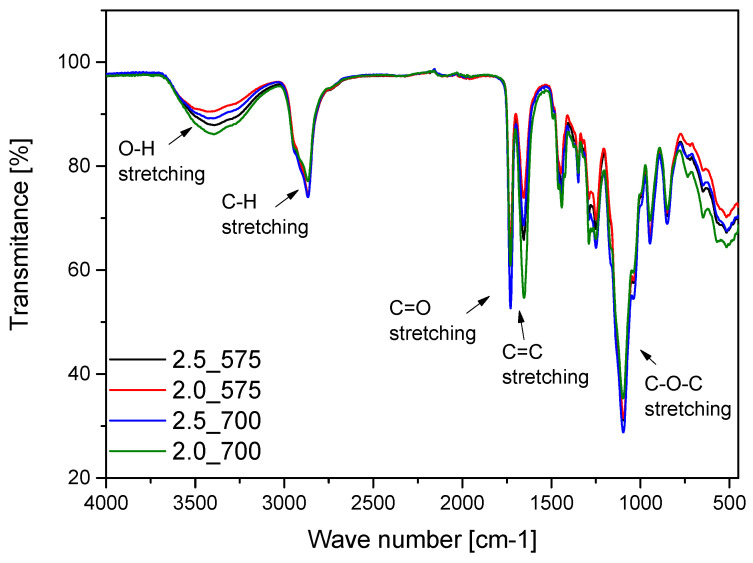
Comparison of absorption bands in FT-IR spectra for samples with varying crosslinking agent content and molecular weight.

Spectroscopic analysis was carried out on samples both before and after incubation. The purpose of this analysis was to determine the effect of incubation on the structure of the materials. The results of this study are shown in Figure 5.

**Figure 6:**
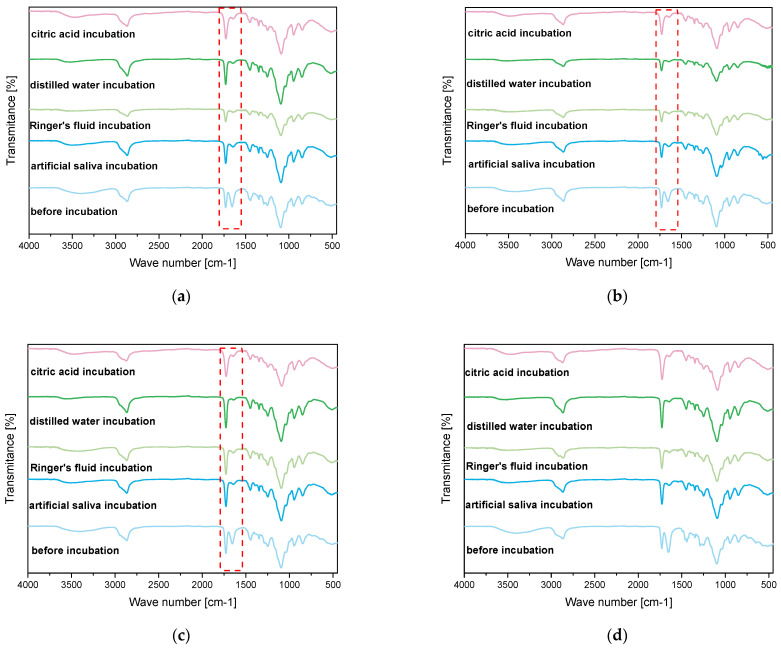
Comparison of FT-IR spectroscopic spectra before and after incubation of sample 2_700 (**a**); 2.5_700 (**b**); 2_575 (**c**); 2.5_575 (**d**).

Infrared spectroscopy (FT-IR) analysis performed on the hydrogel materials enabled the identification of characteristic absorption bands associated with individual components and revealed structural changes during incubation. The result of this analysis are presented in Figure 6. The absorption band corresponding to stretching vibrations of -OH groups was observed in the range of 3380 cm⁻^1^, characteristic of PVA and other hydrophilic components. Additionally, stretching vibrations of C=O bonds appeared prominently at 1650 cm⁻^1^, a feature attributed to PVP and the crosslinking agent PEGDA. The analysis confirmed the presence of C-O stretching vibrations at 1090 cm⁻^1^, derived from PVA, and deformation vibrations of C-H bonds in the range of 1200–980 cm⁻^1^, characteristic of PVP. These functional groups play a crucial role in the chemical stability and hydration properties of the hydrogel matrix, which has been similarly demonstrated in work by Han et al., who observed stability in hydrogels used for tissue engineering applications [20]. Similar findings were reported by Li et al., who studied the structures of hydrogels for the application of alginates. In their study, the induction of OH vibrations was a limiting effect of these groups, which resulted in the hydrogel network methods during incubation [21]. The deformational vibrations in the range of 1200–980 cm⁻^1^ remained largely unchanged, indicating the structural integrity of the hydrogel matrix even after prolonged incubation. Similar findings were made by Guo et al., who observed that the deformational vibrations in the range of 1200–980 cm⁻^1^ remained stable in their study of hemicellulose-based hydrogels. These studies showed that these vibrations, attributed to C-O and C-O-C groups, reflected the structural stability of the hydrogels during mechanical and chemical processing [22]. The structural stability of hydrogels, visualized by FT-IR, is crucial for the use of hydrogel matrices in medical applications. For example, Xin Gan et al. investigated the applications of gelatin-based network hydrogels for cartilage reconstruction. Their study showed that functional groups such as COC and C=O are required for the compatibility and biocompatibility of the materials. These results are consistent with the analysis presented in the data submission. The similarity of the results with studies from the literature emphasizes the importance of the impact and structural integrity of hydrogels under incubation conditions. The preservation of basic functional groups such as -OH, C=O, and COC confirms their suitability for biomedical applications [23].

Despite the significant similarity in FT-IR spectra before and after incubation in simulated body fluids, changes were observed: partial disappearance and a substantial decrease in the intensity of the absorption band at approximately 1650 cm⁻^1^, corresponding to the stretching vibrations of C=O bonds. This phenomenon may result from the hydrolysis of ester bonds present in PEGDA under incubation conditions, leading to partial structural degradation of these groups. Furthermore, the reduction in the intensity of the C=O band could be associated with interactions between the hydrogel and ions present in the simulated body fluids, which may affect the local chemical environment of the carbonyl groups. The interpretation of these results suggests that while the fundamental functional groups of the hydrogel, such as -OH and C-O-C, remain stable, certain structural changes occur within the polymer network due to the influence of simulated biological conditions. These findings highlight the importance of optimizing the chemical composition of hydrogels to ensure their long-term stability in biomedical applications.

### 2.5. Characterization of Hydrogels via Microscopic Techniques

**Figure 7:**
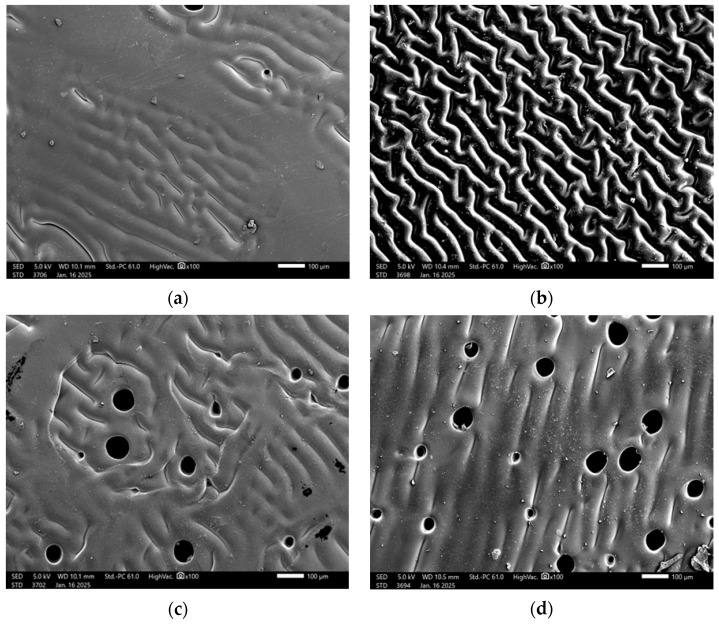
SEM images of hydrogel samples 2_575 (**a**); 2.5_575 (**b**); 2_700 (**c**); 2.5_700 (**d**).

To investigate the structure and properties of the developed materials, observations were carried out using scanning electron microscopy (SEM). Images of samples with varying contents and types of crosslinking agents were compared to analyze their morphological differences. These observations and comparisons are presented in Figure 7.

SEM analysis is employed for imaging and analyzing the surface of samples at the micro- and nanometer scale. It is an advanced research technique used to investigate chemical composition and surface morphology, and to identify defects in materials. The obtained SEM images revealed changes in the surface morphology of dried hydrogels. It was hypothesized that the amount of crosslinking agent used in the photopolymerization process and its molecular weight would significantly affect the surface morphology of the hydrogels. The SEM study results confirmed the proposed hypothesis. The analysis demonstrated the influence of the crosslinking agent and its molecular weight on the hydrogel’s surface. Hydrogel matrices formed with a crosslinking agent of 700 g/mol molecular weight were characterized by a porous structure. The SEM image of the sample with 2 mL of crosslinking agent with a molecular weight of 575 g/mol showed a relatively smooth surface, with the structure being less organized compared to the sample with a higher PEGDA content, which displayed a clearly arranged structure in regular patterns. In this case, increasing the amount of crosslinking agent contributed to the organization of the structure, potentially leading to improved mechanical properties in the sample. Images of the structural morphology for the sample containing 2 mL of the crosslinking agent with a molecular weight of 700 g/mol showed openings in the structure, indicating the porous nature of the matrix. The sample exhibited an uneven pore distribution and a lower degree of surface organization compared to the sample with 2.5 mL of PEGDA with a molecular weight of 700 g/mol. Increasing the amount of the crosslinking agent with a higher molecular weight led to better organization of the polymer network.

### 2.6. Analysis of the Density of Polymeric Materials

**Figure 8:**
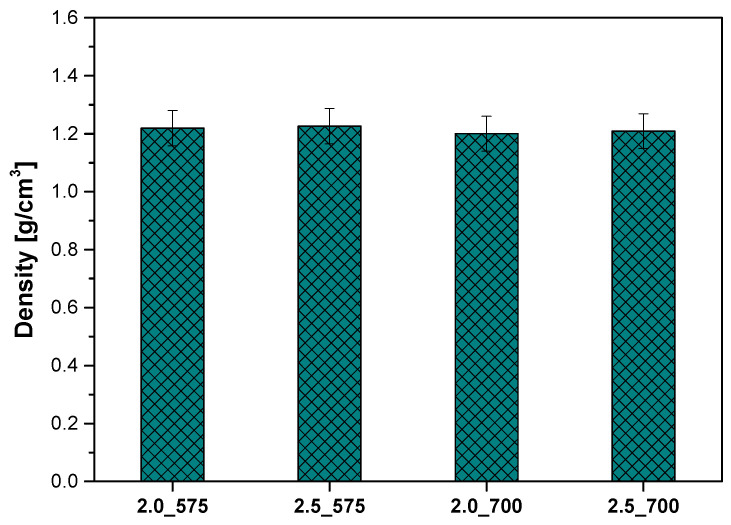
Results of density analysis of polymeric materials.

An analysis of density was conducted on the samples, which differed in the type and quantity of the crosslinking agent. The measurements were performed using a hydrostatic balance based on Archimedes’ principle. The results are presented in the figure below (Figure 8).

It was observed that a higher amount of the crosslinking agent (2.5 mL) resulted in slightly higher density compared to samples containing 2.0 mL of the crosslinking agent, both for the molecular weight of 575 g/mol and 700 g/mol. For example, the density of sample 2.5_575 (1.22602 g/cm^3^) was higher than that of sample 2.0_575 (1.21934 g/cm^3^), and sample 2.5_700 (1.2088 g/cm^3^) exceeded sample 2.0_700 (1.2004 g/cm^3^). The differences in density between the samples are minor, suggesting that changes in the amount and molecular weight of the crosslinking agent have a moderate influence on this parameter. However, in conjunction with findings from other analyses indicating significant differences in other properties, such as surface morphology or roughness, it can be inferred that even slight variations in density may play an important role in shaping the functional properties of hydrogels. These conclusions underscore the importance of a comprehensive analysis of various parameters to fully understand the impact of the crosslinking process on the structure and properties of hydrogels. Previous studies have shown that density alone may not fully capture the functional potential of hydrogels for biomedical applications, where other factors like mechanical strength and the release profile of incorporated bioactive compounds play a crucial role in determining the material’s performance [24]. Thus, it is important to consider density in conjunction with other parameters such as swelling behavior, mechanical properties, and stability in physiological conditions. This comprehensive analysis provides a more accurate assessment of the impact of the crosslinking process on the structure and properties of hydrogels.

### 2.7. Synthesis and Characterization of Polymeric Material Modified with Mixture of Bioactive Compounds

In this study, hydrogels were modified by incorporating a mixture of bioactive compounds, including glucosamine, chondroitin, and methylsulfonylmethane (MSM), in specific concentrations: 125 mg of glucosamine, 100 mg of chondroitin, and 75 mg of MSM. These bioactive molecules are well-known for their regenerative properties, particularly in the context of cartilage tissue repair and regeneration.

The release of bioactive compounds from hydrogels can occur through three primary mechanisms: diffusion, degradation, and swelling. Diffusion is the predominant mechanism responsible for the release of small molecules such as glucosamine and chondroitin. These molecules move from regions of higher concentration inside the hydrogel to lower concentrations in the surrounding environment. Due to their relatively small molecular size, they can easily diffuse through the polymeric matrix. Although MSM is a larger molecule, it also follows the diffusion mechanism, albeit with a slower release rate due to its size [25]. Degradation refers to the breakdown of the hydrogel matrix over time, which releases encapsulated bioactive molecules. Hydrogels with higher crosslinking density degrade more slowly, which limits the contribution of this mechanism to the release of bioactive compounds in the short term. Therefore, degradation plays a secondary role compared to diffusion and swelling in our hydrogels. Swelling occurs when the hydrogel absorbs water, causing it to expand and subsequently release encapsulated bioactive molecules. This mechanism is particularly important for the release of larger molecules like MSM [16]. Swelling leads to a more porous structure, enabling the sustained release of the bioactive compounds from the hydrogel matrix. Based on the observed swelling behavior in our hydrogels, diffusion is expected to be the primary release mechanism for glucosamine and chondroitin, while swelling is anticipated to play a more prominent role in the release of MSM. Under physiological conditions, glucosamine aids in the synthesis of glycosaminoglycans, which are critical for maintaining cartilage structure. Chondroitin sulfate contributes to the mechanical properties of the extracellular matrix, providing structural support to cartilage. MSM offers anti-inflammatory effects, helping to alleviate pain and inflammation associated with cartilage degeneration. These bioactive compounds are integrated into the hydrogel matrix to support cartilage repair and regeneration. The controlled release of these compounds from the hydrogel ensures sustained therapeutic effects over time, promoting long-term healing. Given the swelling capacity and diffusion rates observed in our hydrogels, diffusion will be the dominant release mechanism for glucosamine and chondroitin, while swelling will facilitate the release of MSM, contributing to the overall therapeutic potential of the material.

**Figure 9:**
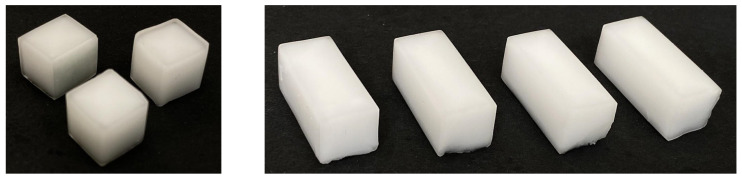
Example images of hydrogel materials modified with bioactive substances.

To assess the potential of the developed polymeric material as a delivery platform for bioactive substances promoting cartilage tissue regeneration, a selected sample, serving as the base material, was modified using a mixture of bioactive compounds. Representative images of the modified materials are presented below in Figure 9.

The FT-IR spectrum of the base hydrogel was discussed above in Section 2.4. During the analysis of the modified hydrogel, this spectrum was revisited for better interpretation. This spectrum is characteristic of the polymeric components PVA and PVP, which form the basis of the developed material. Subsequently, the spectrum for the bioactive component, a mixture of glucosamine, chondroitin, and MSM, reveals the presence of several key bands associated with its chemical structure. A broad band appears in the range of 3200–3500 cm⁻^1^, associated with hydroxyl (-OH) and amino (-NH_2_) groups derived from glucosamine and chondroitin. Signals in the range of 2800–3000 cm⁻^1^ correspond to the stretching of C-H bonds in alkyl groups. A prominent band around 1400–1500 cm⁻^1^ can be attributed to sulfate groups characteristic of chondroitin, while bands in the range of 1000–1100 cm⁻^1^ are linked to C-S and S=O bonds, typical of MSM. The observed bands confirm the chemical structure of the bioactive component and its features, such as sulfate and sulfoxide groups.

**Figure 10:**
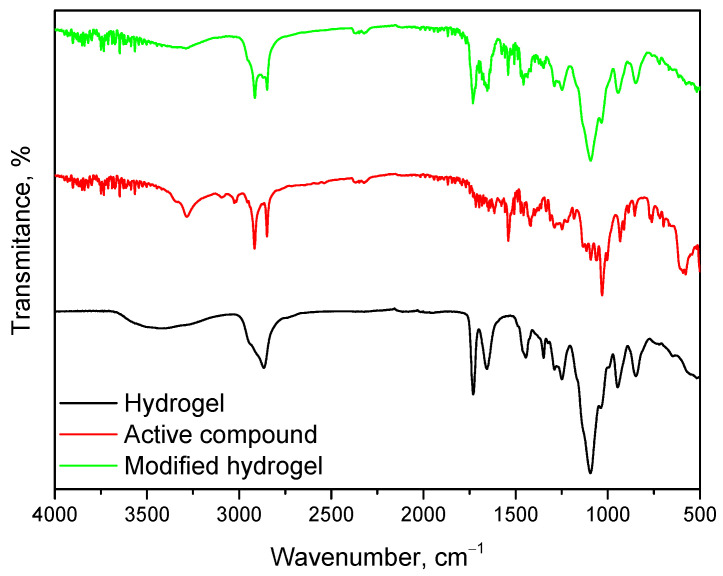
FT-IR spectroscopy spectra of hydrogel material, active compound, and modified material.

The spectrum of the hydrogel modified with the bioactive component exhibits characteristics of both the base hydrogel and the bioactive component, indicating successful modification (Figure 10). The broad band in the range of 3200–3500 cm⁻^1^ becomes more pronounced, reflecting an increased number of hydroxyl and amino groups derived from the bioactive component. Additionally, more prominent bands in the range of 1400–1500 cm⁻^1^ confirm the presence of sulfate groups characteristic of chondroitin. Possible changes in intensity or shifts in the range of 1650–1750 cm⁻^1^ suggest interactions between the carbonyl (C=O) groups of the PVP polymer and the bioactive components, which may indicate the formation of hydrogen bonds or electrostatic interactions between the hydrogel matrix and the bioactive mixture. Such a coherent spectral profile unequivocally confirms the successful integration of the bioactive component into the hydrogel matrix while preserving the distinctive features of both components. The conducted analyses indicate that the modified hydrogel can effectively serve as a carrier for the mixture of glucosamine, chondroitin, and MSM, potentially enhancing its bioactive properties. The FT-IR spectra confirm the presence of active ingredients in the hydrogel and the potential for chemical interactions that may influence its mechanical properties and biocompatibility. It is proposed that such systems could find applications in regenerative medicine, particularly in the treatment of joint diseases and cartilage tissue repair.

**Figure 11:**
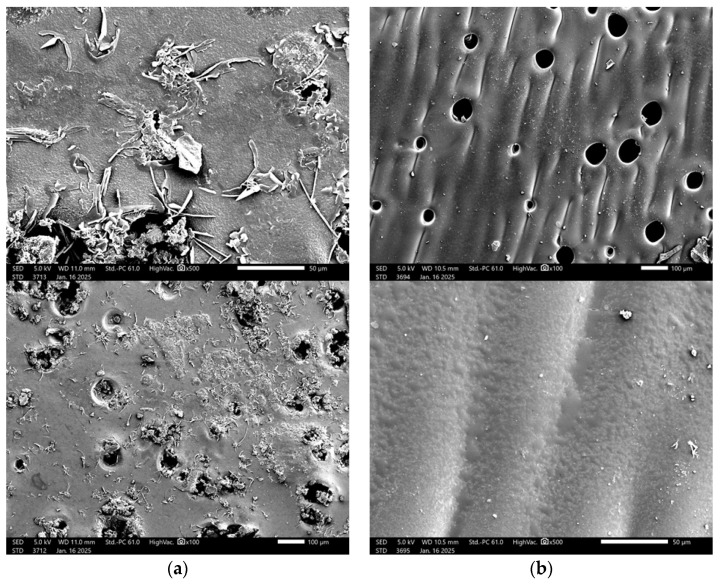
SEM images of the sample after modification with bioactive compound (**a**) and the sample before modification (**b**).

Based on the presented SEM images, a comparative analysis was conducted between the unmodified hydrogel sample (Figure 11b) and the biologically modified sample (Figure 11a). The surfaces of both samples exhibit a porous structure, which is characteristic of hydrogels crosslinked using 2.5 mL of a crosslinking agent with a molecular weight of 700 g/mol. However, the biologically modified sample (Figure 11a) is distinguished by a greater number of irregularities and additional surface roughness, likely resulting from the incorporation of bioactive compounds such as chondroitin, glycosaminoglycans, and MSM. The introduction of these compounds may have caused local reorganization or modification of the polymer network’s topology, leading to a more complex and heterogeneous surface structure. In contrast, the unmodified sample (Figure 11b) displays a more homogeneous surface with fewer visible irregularities, indicating the absence of additional factors influencing the morphology of the polymer network. The observed differences may have significant implications for potential biomedical applications. The more complex surface morphology of the biologically modified sample (Figure 11a) may enhance cell adhesion and interactions with the biological environment, potentially increasing the material’s bioactivity and regenerative capabilities. Meanwhile, the more uniform structure of the unmodified sample may be advantageous in applications requiring greater surface stability. Ultimately, the biological modification process demonstrates a significant impact on the structural properties of hydrogels, underscoring its importance in tissue engineering, particularly for cartilage regeneration. By using a bioactive compound, we can have a beneficial effect on native cartilage by increasing the similarity of the material to cartilage tissue cells. The modification can be adapted to reproduce the properties of native cartilage in terms of elasticity and strength, can create a favorable environment for cell growth, and can also act as a controlled release system, which can affect the processes of real tissues and regeneration. Additionally, it is worth emphasizing that native cartilage does not have the ability to self-regenerate, which gives potential for polymer applications. Similar conclusions were drawn by Yuriy Melnyk et al. in their research on hydrogel membranes for encapsulated forms of drugs. In their work, they emphasize that chemical modification of the hydrogel membrane by graft copolymerization is a possibility to improve the properties of the polymer [26].

## 3. Materials and Methods

### 3.1. Materials

Poly(vinyl alcohol) (PVA, crystalline powder, 87–89% hydrolyzed, Mw 13,000–23,000), polyvinylpyrrolidone (PVP, powder, average mol wt. 10,000), diacrylate poly(ethylene glycol) (crosslinking agent, PEGDA, average molecular weight Mn = 700 g/mol and Mn = 575 g/mol), and 2-hydroxy-2-methylpropiophenone (photoinitiator, 97%, d = 1.077 g/mL) were purchased from Sigma Aldrich (Saint Louis, MO, USA). The combination of bioactive compounds was purchased as a dietary supplement from SWANSON (West Auckland, New Zealand) with the following composition: Glucosamine/Chondroitin/MSM = 250 mg/200 mg/150 mg.

### 3.2. Synthesis of Hydrogel Materials

The first step in the synthesis of hydrogel materials involved the preparation of various polymer solutions: polyvinylpyrrolidone (PVP) at a concentration of 15% and poly(vinyl alcohol) (PVA) at a concentration of 5%. The respective amounts of these solutions were then taken and mixed in the specified order. Appropriate amounts of the crosslinking agent, poly(ethylene glycol) diacrylate (PEGDA), were added to the polymer mixture, and finally, the photoinitiator, which was 2-hydroxy-2-methylpropiophenone, was added. The mixture was transferred to a Petri dish and placed under a UV lamp (model EMITA VP-60, power 180 W, wavelength λ = 320 nm, Famed-1, Łódź, Poland). The sample was irradiated for 120 s. The composition of each hydrogel is shown in Table 2.

### 3.3. Sorption Capacity Analysis

The analysis aimed to investigate the ability of hydrogel matrices to swell in detail, which is crucial for their use in regenerating cartilage tissue. The study was conducted to assess whether hydrogel materials could effectively absorb therapeutic fluids, which could significantly improve cartilage regeneration. Pre-prepared and dried hydrogel disks were used, which provided a representative model for such studies. First, each sample was accurately weighed on an analytical balance to ensure the precision of the measurements and then placed in four different incubation fluids: distilled water, citric acid, Ringer’s fluid, and saliva. These fluids were chosen to simulate the different conditions that can occur in a medical environment. Samples were incubated in these fluids for specific periods of time, 1 h, 24 h, and 12 days, to investigate the short-term and long-term effects of swelling. After each incubation period, excess fluid was removed from the surface of the disks to ensure the accuracy of the weight measurements, which were again measured on a Radwag analytical balance. Finally, the average swelling index values obtained from repeated trials were used to determine the sorption capacity of the hydrogel matrices by determining the sorption coefficient *α*.

(1) $$\alpha =\frac{\left(m_{t}-m_{0}\right)}{m_{0}}$$

where *α*—swelling ratio, g\g; *m_t_*—mass of swollen sample after time “*t*”, g; *m*_0_—mass of dry sample (before the study), g.

### 3.4. Incubation Studies

The incubation study was designed to investigate how the hydrogel matrices interact with solutions imitating human physiological fluids. Changes in pH values indicated the leaching of uncross-linked agents or caused degradation of samples in fluids, among other things. Samples with a diameter of 1 cm were sterilely placed in containers containing 50 mL each of incubation fluids: distilled water, citric acid, Ringer’s solution, and saliva. Samples were incubated at 37 °C and pH was measured for 28 days using a CX-701 ELMETRON multifunctional device (Elmetron, Zabrze, Poland).

### 3.5. Microscopic Observations and Surface Roughness Profile Analysis

A high-technology digital microscope, the VKX-7000 from Keyence (Osaka, Japan), capable of accurately producing images at 4 K resolution, was used. The microscope was equipped with an advanced CEO REMAX optical engine and a 4 K CMOS image sensor, which provided high accuracy and magnification. The main objective was to study the surface morphology of hydrogels and determine their roughness profiles.

### 3.6. Infrared Spectroscopy Analysis

To identify the characteristic functional groups in the hydrogel structure, FT-IR spectroscopy was used. A Nicolet iS5 Thermo Scientific spectrometer (Loughborough, UK) was used for the analysis and measurements were performed at room temperature. FT-IR spectra were recorded between 4000 and 500 cm^−1^, taking 32 scans at a resolution of 4.0 cm^−1^. Both incubated and non-incubated samples were analyzed to assess the potential degradation of the material.

### 3.7. Analysis of the Density of Polymeric Materials

The density of the hydrogel samples was determined using a density measurement kit specifically designed for RADWAG balances. The measurements were conducted at room temperature, utilizing isopropanol as the immersion liquid. Five measurements were performed for each sample, and the final density value was calculated as the mean of these measurements, ensuring the accuracy and reliability of the results.

### 3.8. Characterization of Hydrogels via Microscopic Techniques

SEM analysis was performed for hydrogel samples that had been dried and sputtered with a layer of nanogold. The observations were made using a scanning electron microscope JOEL IT200 (JEOL Ltd., Peabody, MA, USA).

### 3.9. Synthesis and Characterization of Polymeric Material Modified with Mixture of Bioactive Compounds

In order to check the potential of the obtained hydrogel materials in cartilage tissue regeneration, synthesis was carried out with a mixture of bioactive compounds (glucosamine, chondroitin, methylsulfonylmethane). This synthesis was carried out analogously to the synthesis described in Section 3.2, but only one sample containing the largest amount of a cross-linking agent with a higher molecular weight (2.5 (700)) was selected. During the synthesis, a mixture of bioactive compounds was added in the following amounts: glucosamine 125 mg, chondroitin 100 mg, methylsulfonylmethane 75 mg. Fourier infrared spectroscopy (FTIR) analysis was performed for the obtained hydrogel matrices.

## 4. Conclusions

- The swelling capacity of hydrogels is significantly influenced by the amount and molecular weight of PEGDA. Lower amounts of PEGDA and lower molecular weight (575 g/mol) resulted in higher swelling coefficients due to a looser network structure, while higher amounts of PEGDA with higher molecular weight (700 g/mol) produced denser networks with reduced water absorption.
- Increased amounts of PEGDA lead to higher surface roughness both before and after incubation, with significant changes observed in samples containing PEGDA 575 g/mol, highlighting their susceptibility to structural alterations. PEGDA 700 g/mol provided better stability and resistance to degradation.
- The pH stability of the hydrogels was largely unaffected by incubation in distilled water and citric acid, confirming the absence of degradation. However, a significant pH jump was observed in artificial saliva, likely due to interactions with its complex composition, without evidence of hydrogel degradation. Such interactions underscore the importance of the chemical environment in determining hydrogel performance.
- FT-IR spectroscopy confirmed the presence of characteristic functional groups of both the hydrogel matrix and bioactive mixture. The integration of bioactive components (glucosamine, chondroitin, MSM) was successful, as indicated by new and intensified absorption bands. These modifications suggest potential improved bioactivity and compatibility.
- The study demonstrates that the developed hydrogels, particularly those modified with bioactive compounds, have significant potential for applications in regenerative medicine, such as joint disease treatment and cartilage repair, due to their customizable properties, biocompatibility, and chemical stability. Further studies are recommended to optimize their properties.

## Figures and Tables

**Table 1:** Roughness parameters for hydrogel materials.

Sample Name	Ra [µm]	Rz [µm]
2_575	2.43	17.42
2.5_575	14.32	153.47
2_700	5.12	25.52
2.5_700	13.42	80.20

**Table 2:** Composition of hydrogel materials.

15% PVP 10 k [mL]	5% PVA 13–23 k [mL]	PEGDA [mL]	Photoinitiator [µL]	Sample Name *
3	7	2 (700)	50	2_700
		2.5 (700)		2.5_700
		2 (575)		2_575
		2.5 (575)		2.5_575

* The sample names correspond to the volume of the crosslinking agent used, expressed in mL, followed by its average molecular weight in g/mol.

## Data Availability

The original contributions presented in this study are included in the article. Further inquiries can be directed at the corresponding authors.
